# Editorial: Community series in caring for those who are neglected and forgotten: psychiatry in prison environments, volume II

**DOI:** 10.3389/fpsyt.2024.1549139

**Published:** 2025-01-10

**Authors:** Norbert Konrad, Annette Opitz-Welke, Birgit Völlm

**Affiliations:** ^1^ Institute for Forensic Psychiatry, Charité University Medicine Berlin, Berlin, Germany; ^2^ Department of Forensic Psychiatry, University Hospital Rostock, Rostock, Germany

**Keywords:** prison, self-harm, suicide, Ethiopia, enuresis, release

Individuals in prison have an increased risk of suffering from mental disorders, including substance use disorders, as well physical health challenges (1). Suicide rates in penal institutions are several times higher than that of the general population and suicide is the leading course of death in prison (2). The prevalence of self-harm in prisoners is also high at about 5% in males and up to 25% in females (3) presenting the often poorly resourced prison health care services with significant challenges. Standards of screening and care for mentally disordered prisoners as well as the management of transition back into the community are a matter of ongoing debate in policy and research. This Research Topic is the second on this population demonstrating ongoing interest from researchers in this field (see the first volume here).

Of the seven papers included in this volume three are on the topic of self-harm or suicide indicating again the need to provide evidence on these topics in order to improve their management and prevent further harm through imprisonment. Hausam et al. address the important topic of screening for risk of suicide. They compared two different screening instruments in male prisoners in the Berlin Prison System, the Screening for Initial Risk Assessment (SIRAS) and the Vienna Instrument for Suicidality with the former identifying high-risk prisoners more reliably. The agreement of the two instruments was poor indicating the need for further research as well as careful consideration when choosing instruments for specific settings.

Blees et al. present a pilot study aiming to describe the characteristics of prisoners who self-harm in the Berlin Prison System. Although based on a small non-representative sample, the study provides important information which could serve to inform preventive strategies. Those who self-harmed differed from the general prison population in that younger prisoners, women and those with migration backgrounds were overrepresented. Almost all of those who self-harmed had a psychiatric diagnosis, predominantly substance use disorders in men and borderline personality disorder in women. About two-thirds of self-harm incidents occurred on remand, the most common method was cutting. Importantly, nearly half of the sample were on special security measures during the period of self-harm, mainly solitary confinement, calling for alternative, psychosocial, supportive rather than security interventions to support those at risk of self-harm.

Suicide in prisoners does not only affect the person themselves and their families but also those in the vicinity of the incident, namely other prisoners and staff. Mental health staff caring for a person who subsequently commits suicide are particularly affected but the impact on staff in a prison setting specifically is poorly understood. Fontao et al.’s review is therefore a welcome summary of our knowledge on this topic to date. Despite the high relevance, the authors only identified six empirical research papers, one quantitative and five qualitative. highlighting the urgent need for more attention to the impact of prison suicide in order to inform support structures for staff. This is even more important as the psychological effect on staff appears to be high - e.g. over one third of prison officers scored high on a trauma symptom inventory and qualitative studies supported a high prevalence of mental health consequences and limited support available to those affected.

Kös et al. present another study comparing two instruments, this time regarding violence towards others in a sample of involuntarily admitted patients in Germany. They showed that the German version of the Violence Risk Screening-10 (V-RISK-10) was better in predicting longer term outcomes while the Brøset Violence Checklist (BVC) predicted short term violence more reliably. The authors concluded that structured assessment instruments may be useful in this group of patients but caution to solely rely on such actuarial instruments as they do not allow to take into consideration individual factors and might therefore disadvantage certain individuals.

Koposov et al. explored the prevalence of enuresis in incarcerated young offenders. They found a prevalence of 20% of historical and 10% of current enuresis. Those with enuresis showed no differences compared to offending youth in terms of other diagnoses but had higher rates of self-reported mental health problems as well as self-harm. The authors suggest particular attention should be paid to risk to self in those identified with enuresis at health screening for young offenders.

The post-release period is one with increased risk, particularly of suicide. It is therefore essential that aftercare is planned well prior to release. Walsh et al. explored how remand prisons in Ireland managed this aftercare planning as well as planning for transfer to another prison or healthcare setting. It is encouraging that they found that successful transition can be achieved. In 90% of those prisoners referred to mental health teams in other prisons face to face contact with a medical professional was achieved within an average of 6 days, for those released into the community this was the case for 60% with an average of 9 days after release. Multi-agency working, in particular the involvement of housing support, increased the likelihood of successful mental health support post-release.

Finally, it is encouraging to see that research in prison populations is also taking place in socioeconomically disadvantaged countries. Yesuf at al. studied health care utilization in three prisons in Ethiopia and identified factors associated with higher use. These included information about services available, higher education and sentenced (as opposed to remand) status. Overall over 70% of prisoners used medical but only 13.3% psychiatric services. This might be related to the stigma associated with mental health conditions in Ethiopia and should be further explored in future research.

We hope that the findings of this research will translate into tangible changes in the clinical care for this neglected group.
